# A development of machine learning models to preoperatively predict insufficient clinical improvement after total knee arthroplasty

**DOI:** 10.1186/s13018-025-06206-z

**Published:** 2025-08-20

**Authors:** Geunwu Gimm, Byoungjun Jeon, Sung Eun Kim, Byeong Soo Kim, Hyuk-Soo Han, Sungwan Kim

**Affiliations:** 1 Department of Biomedical Engineering, Seoul National University College of Medicine, 103 Daehak-ro, Jongno-gu, Seoul, 03080 Republic of Korea; 2 Department of Orthopaedic Surgery, Seoul National University College of Medicine, 103 Daehak-ro, Jongno-gu, Seoul, 03080 Republic of Korea; 3 Department of Orthopaedic Surgery, Seoul National University Hospital, 101 Daehak-ro, Jongno-gu, Seoul, 03080 Republic of Korea; 4 Office of Hospital Information, Seoul National University Hospital, 101 Daehak-ro, Jongno-gu, Seoul, 03080 Republic of Korea; 5 Interdisciplinary Program in Bioengineering, Graduate School, Seoul National University, 1 Gwanak-ro, Gwanak-gu, Seoul, 08826 Republic of Korea

**Keywords:** Total knee arthroplasty, Insufficient clinical improvement prediction, WOMAC, Minimal clinically important difference, Machine learning

## Abstract

**Background:**

Identifying patients unlikely to achieve meaningful improvement following total knee arthroplasty (TKA) supports more effective shared decision-making (SDM). This study aimed to develop and validate machine learning (ML) models that preoperatively predict insufficient clinical improvement one year after TKA using Western Ontario and McMaster Universities Osteoarthritis Index (WOMAC) subscales and total scores, and to assess the important predictive variables.

**Methods:**

A retrospective analysis was performed on consecutive primary TKA patients from 2004 to 2022 at a single tertiary hospital was conducted. Insufficient clinical improvement was defined as not achieving the minimal clinically important difference (MCID) for each WOMAC subscale and total. Candidate preoperative variables included demographics, comorbidities, knee range of motion, radiologic variables, and WOMAC scores. A variety of ML models were evaluated using performance metrics for calibration and discrimination, as well as decision curve analysis and Shapley additive explanations.

**Results:**

Among the 3,810 TKAs included, the ExtraTrees model performed best for WOMAC pain, stiffness, function, and total scores, achieving AUCs of 0.92, 0.90, 0.87, and 0.89; recall rates of 0.79, 0.86, 0.70, and 0.83; and Brier scores of 0.09, 0.10, 0.11, and 0.06, respectively, along with demonstrating good calibration curves and net clinical benefit. Shapley additive explanations identified better preoperative WOMAC scores, osteoporosis, diabetes mellitus, older age, malignancy, and coronary artery disease as important predictors of insufficient clinical improvement.

**Conclusions:**

The ML models demonstrated good performance in preoperatively predicting insufficient clinical improvement at 1 year after TKA based on WOMAC. These models have the potential to enhance SDM and perioperative patient management by preoperatively identifying approximately 70% to over 80% of patients likely to experience insufficient clinical improvement, with a specificity of about 80%, and by providing explanations regarding associated factors.

**Supplementary Information:**

The online version contains supplementary material available at 10.1186/s13018-025-06206-z.

## Background

Shared decision-making (SDM) is a collaborative approach that involves clinicians and patients in determining the optimal treatment for each specific case [1, 2]. Recently, SDM has emerged as a core principle in clinical decision-making and is being increasingly implemented for interventions such as total knee arthroplasty (TKA), with support from organizations such as the American Academy of Orthopaedic Surgeons (AAOS) [3]. One of the key elements of SDM [1, 3] is discussing expected improvements after TKA to clarify options and systematically weigh potential harms and benefits.

TKA is the most commonly performed surgery for severe knee osteoarthritis and is widely recognized as a cost-effective intervention with a satisfactory success rate [4–6]. While earlier reports indicated a 20% dissatisfaction rate following TKA, more recent studies suggest this rate has declined to 10% [7–11]. As patient satisfaction rates have increased, identifying patients at risk of dissatisfaction has become a more important and challenging task. Accurately identifying individuals unlikely to achieve meaningful improvement after TKA could help guide discussions about surgical decision-making, optimize perioperative management, and refine patient expectations, thereby improving the efficacy of SDM in clinical practice.

Numerous studies have aimed to predict postoperative satisfaction or whether scores on patient-reported outcome measures (PROMs) would achieve the minimal clinically important difference (MCID) after TKA, and these efforts have shown acceptable performance [8, 12–16]. With the advancement of machine learning (ML), recent studies applying ML to these predictions have demonstrated performance comparable to that of traditional statistical approaches [17–24]. Although the Western Ontario and McMaster Universities Osteoarthritis Index (WOMAC) is one of the most widely used PROMs, to our knowledge, no studies have applied ML to predict whether improvement in each WOMAC subscale and total score will not achieve their respective MCIDs, or to explore the extent and direction of associations between predictive variables and specific subscales.

This study aimed to develop and validate ML models to preoperatively predict whether one-year improvements in WOMAC pain, stiffness, function, and total scores following TKA would not exceed their respective MCIDs, and to identify important predictive variables.

## Methods

### Patient enrollment

This retrospective study received approval from the Institutional Review Board and Big Data Review Board of our hospital (H-2208-124-1353). It included consecutive primary TKAs performed for degenerative knee osteoarthritis by four experienced knee surgeons at a single tertiary hospital from December 2004 to March 2022 with a minimum one-year follow-up (*n* = 7,508). Revision TKA, unicompartmental knee arthroplasty, primary TKAs for septic arthritis, rheumatoid arthritis, tuberculosis arthritis, or post-traumatic arthritis were not included. The indications for TKA included severe knee pain unresponsive to conservative treatment and radiologically confirmed high-grade osteoarthritis. All four surgeons applied the mechanical alignment technique. Of the initial 7,508 knees, 3,698 knees were excluded due to the absence of PROM data. As a result, a total of 3,810 knees were analyzed in the study.

### Data collection

Demographic variables and comorbidity data, including coronary artery disease, congestive heart failure, arrhythmias, peripheral vascular disease, cerebrovascular disease, chronic pulmonary disease, diabetes mellitus, liver disease, severe chronic kidney disease, any malignancy [25], osteoporosis, and mental and behavioral disorders (ICD-10 codes F00-F99), as well as preoperative knee range of motion were obtained from electronic medical records. Severe chronic kidney disease was characterized as stage IV or V, corresponding to a glomerular filtration rate below 29 ml/min/1.73m^2^ [26]. Preoperative radiologic variables, including the hip-knee-ankle angle (HKAA), medial proximal tibial angle (MPTA), and Kellgren-Lawrence grade (K-L grade), were assessed using a deep learning model that had previously demonstrated robust performance [27]. The WOMAC scores were collected preoperatively and 1 year postoperatively. A follow-up period of 1 year was chosen because prior studies have shown that notable improvements in pain, function, and mental health reach a plateau at this time after TKA [15, 28]. The WOMAC, a widely used PROM for TKA, recognized for its quality [29, 30], comprises 24 questions across three subscales: pain (5 questions), stiffness (2 questions), and function (17 questions). Each question is scored using a 5-point Likert scale, from 0 to 4 points, with higher scores reflecting greater impairment [31, 32]. Improvement was defined as the difference between the preoperative and one-year postoperative scores, where positive values denoted improvement. Missing data were present for knee range of motion, HKAA, MPTA, K-L grade, and WOMAC scores in 7.1% of the dataset. To address missing data, mean imputation was implemented, whereby each missing value was replaced with the mean of the corresponding variable calculated from non-missing values. This approach was selected to maintain the sample size while preserving the original variable distributions.

### Outcomes

The models were developed to preoperatively predict whether the improvement in each WOMAC score at one year after TKA would not achieve the respective MCID, reflecting insufficient clinical improvement. Important predictive variables were also identified.

The MCID, the minimum difference perceived as beneficial after TKA [31, 33], was adopted from previous research using anchor-based methods: WOMAC pain subscale at 6.7, stiffness subscale at 1.7, function subscale at 13.3, and total at 14.4 (Table 2) [32, 33]. The primary source study [32] derived these MCIDs from a TKA population with clinical characteristics—such as demographics, primary diagnosis, and follow-up duration—comparable to those of our cohort.

### Candidate variables

Candidate variables were chosen from preoperative data to support preoperative decision-making on proceeding with surgery, drawing on previous research indicating that intraoperative or in-hospitalization information does not improve prediction of insufficient clinical improvement [19]. The included candidate variables were: age, sex, side, body mass index (BMI), degree of flexion contracture, degree of flexion, K-L grade, HKAA, MPTA, coronary artery disease, congestive heart failure, arrhythmias, peripheral vascular disease, cerebrovascular disease, chronic pulmonary disease, diabetes mellitus, liver disease, severe chronic kidney disease, any malignancy, osteoporosis, mental and behavioral disorders, and the WOMAC domains of pain, stiffness, function, and total.

### Machine learning algorithms and data analysis

A variety of ML algorithms were implemented, covering both traditional linear models and more sophisticated non-linear methods such as ensemble techniques and neural networks, to enable comprehensive and robust predictive modeling. The algorithms included ExtraTrees, Gaussian Naive Bayes, K-Nearest Neighbors, Logistic Regression, and Multi-Layer Perceptron. We chose ML models alongside traditional multivariable statistical analysis due to their inherent ability to capture complex, non-linear relationships and high-order interactions among numerous preoperative variables without requiring pre-specified assumptions such as linearity, independence, or distributional normality [34]. Moreover, while traditional statistical analysis focuses on inferring relationships between variables, ML aims to make predictions as accurate as possible [35].

The dataset was partitioned into training (80%) and test (20%) sets with stratification based on the target variable to preserve class proportions across sets. To ensure methodological rigor and reproducibility, model development involved a 5-fold stratified cross-validation on the training set, during which hyperparameters were systematically tuned using grid search to optimize the model’s ability to identify patients unlikely to achieve the MCID. The complete hyperparameter grids are provided in Appendix Table 1. The tuned model was subsequently evaluated on the test set using performance metrics, including calibration and discrimination, and decision curve analysis (DCA). For sex-based subgroup analysis, the tuned model trained on the combined dataset of both sexes was separately evaluated on the test sets for each sex.

Discrimination refers to the model’s ability to correctly distinguish between patients who will and will not achieve the MCID. The area under the receiver operating characteristic curve (AUC) provides a quantitative assessment. The threshold for classification was determined using Youden’s J statistic, optimizing the trade-off between recall and specificity. Recall, or sensitivity, measures the proportion of true positive predictions among patients who did not achieve the MCID.

Calibration determines how closely the model’s predicted probabilities correspond to the actual observed event rates across the data spectrum. Visual inspection of observed versus predicted probability plots was used as the primary assessment, with good calibration reflected by a close alignment, thus minimizing calibration error. Its quantitative measures included the Brier score and maximum calibration error (MCE). The Brier score, ranging from 0 (indicating perfect calibration) to 1 (indicating the worst calibration), assesses both calibration and discrimination [17, 18, 36].

To evaluate the clinical utility of each prediction model, DCA was performed. DCA is a method for quantifying the net benefit of the prediction model across a range of risk thresholds, thereby assessing whether its application in clinical practice would result in more benefit than harm. For example, in this study, the prediction model aims to identify patients unlikely to achieve MCIDs after TKA, where accurate identification (true positives) may enable personalized perioperative strategies or help avoid TKA. However, because the model’s specificity is not perfect, there remains a risk that some patients who would have benefitted from TKA might be incorrectly classified and potentially denied TKA or subjected to unnecessary perioperative interventions. Thus, net benefit was calculated as: $\:\frac{True\:positives}{N}-\frac{False\:positives}{N}\times\:\frac{{p}_{t}}{1-{p}_{t}}$, where N is the total sample size and p_t_ is threshold probability [37]. The threshold probability represents the minimum level of predicted risk at which a clinician or patient would decide to proceed with an intervention. Two default strategies, intervening in all patients or in none, were used as references for comparison. A model was considered to have clinical utility if it provided a higher net benefit than both default strategies [38]. The best-performing model was chosen based on calibration and discrimination metrics, as well as DCA.

Shapley additive explanations (SHAP) with summary plots were utilized to provide insights into the contributions of each variable to the model’s predictions. Within the summary plot, variables were displayed vertically in order of importance, and each SHAP value was depicted as horizontally positioned dots. The color spectrum of dots ranged from blue (low) to red (high). The presence of red dots on the right side and blue dots on the left indicated an increased risk with rising variable values [39].

The entire process was conducted using Python, using libraries such as pandas for data manipulation, scikit-learn for model development and evaluation, and SHAP for interpreting model predictions.

## Results

### Baseline characteristics

Table 1 presents the baseline characteristics of the 3,810 TKAs. The cohort had a mean age of 70.4 ± 6.4 years, and 90.3% were women. The preoperative flexion contracture averaged 8.9º ± 7.4º and the further flexion angle was 127.9º ± 29.2º. The mean preoperative HKAA was 170.6º ± 6.2º, indicative of predominantly varus alignment. Comorbidities included coronary artery disease (7.8%), cerebrovascular disease (4.0%), diabetes mellitus (20.8%), severe chronic kidney disease (3.4%), malignancy (6.2%), and osteoporosis (7.8%).

**Table 1:** Baseline characteristics

Characteristic	Value
N	3810
Age (years)	70.4 ± 6.4
Sex (%), women | men	90.3 | 9.7
Side (%), right | left	48.7 | 51.3
Body mass index (kg/m2)	26.6 ± 3.6
Preoperative degree of flexion contracture (deg)	8.9 ± 7.4
Preoperative degree of flexion (deg)	127.9 ± 29.2
Preoperative K-L grade (%), 0 | 1 | 2 | 3 | 4	0 | 0 | 1.9 | 28.5 | 69.6
Preoperative hip-knee-ankle angle (deg)	170.6 ± 6.2
Preoperative medial proximal tibial angle (deg)	85.8 ± 3.1
Coronary artery disease (%), yes | no	7.8 | 92.2
Congestive heart failure (%), yes | no	1.3 | 98.7
Arrhythmias (%), yes | no	4.3 | 95.7
Peripheral vascular disease (%), yes | no	1.5 | 98.5
Cerebrovascular disease (%), yes | no	4.0 | 96.0
Chronic pulmonary disease (%), yes | no	3.5 | 96.5
Diabetes mellitus (%), yes | no	20.8 | 79.2
Liver disease (%), yes | no	4.3 | 95.7
Severe chronic kidney disease (%), yes | no	3.4 | 96.6
Malignancy (%), yes | no	6.2 | 93.8
Osteoporosis (%), yes | no	7.8 | 92.2
Mental and behavioral disorders (%), yes | no	4.6 | 95.4

K-L grade, Kellgren-Lawrence grade

### Improvements in the WOMAC scores

Table 2 presents both preoperative and postoperative WOMAC scores along with their corresponding MCIDs. One year after TKA, the mean improvements in WOMAC pain, stiffness, function, and total scores were 8.40 ± 4.04, 2.84 ± 2.30, 23.93 ± 14.73, and 35.17 ± 18.86, respectively, each exceeding its MCID. The proportions of TKAs that did not achieve the MCIDs were 29.69%, 23.70%, 23.36%, and 12.62%, respectively.

**Table 2:** Preoperative and 1-year postoperative WOMAC scores and score improvements of the study population

Variable	Preoperative score, Mean (SD)	1-year postoperative score, Mean (SD)	Improvement, Mean (SD)	MCID	Proportion of TKAs not achieving MCID (%)
WOMAC pain	9.65 (3.74)	1.25 (2.16)	8.40 (4.04)	6.7	29.69
WOMAC stiffness	3.95 (2.02)	1.11 (1.24)	2.84 (2.30)	1.7	23.70
WOMAC function	35.60 (12.17)	11.67 (9.00)	23.93 (14.73)	13.3	23.36
WOMAC total	49.20 (16.16)	14.03 (10.96)	35.17 (18.86)	14.4	12.62

WOMAC, Western Ontario and McMaster Universities osteoarthritis index; SD, standard deviation; MCID, minimal clinically important difference

### Model performance: discrimination and calibration

**Fig. 1:**
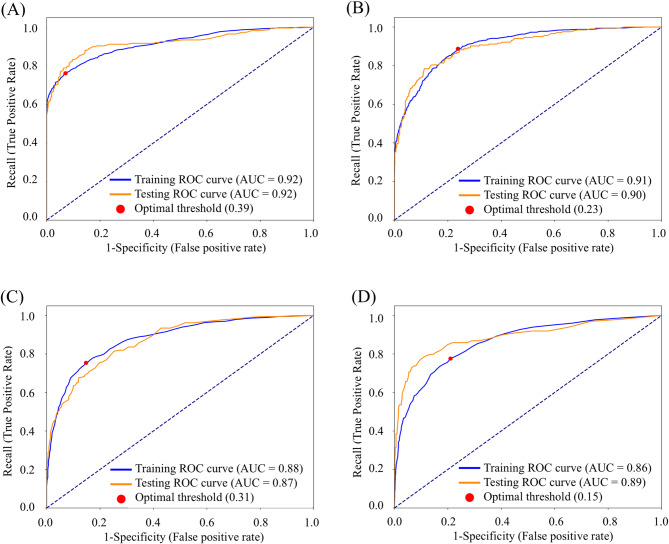
ROC curves (solid lines) with AUC values and optimal threshold (dot) for the best-performing models; the blue line indicates the training data set and the orange line represents the test data set. The optimal threshold was determined by applying Youden’s J statistic to achieve an optimal balance between recall and specificity **(A)**, WOMAC pain; **(B)**, WOMAC stiffness; **(C)**, WOMAC function; **(D)**, WOMAC total. ROC, receiver operating characteristic; AUC, area under the ROC curve; WOMAC, Western Ontario and McMaster Universities Osteoarthritis Index

**Fig. 2:**
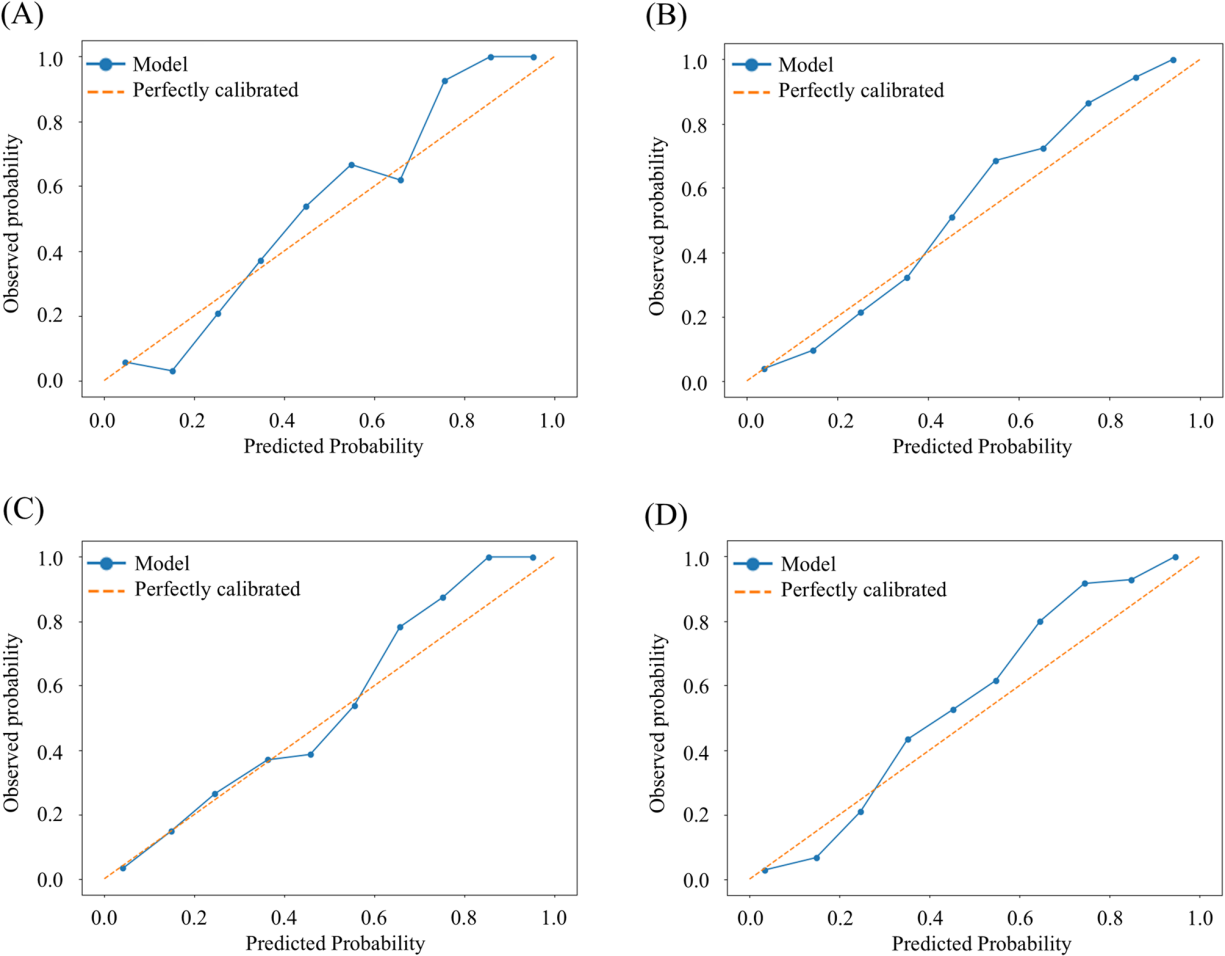
Calibration curves for the best-performing models. The solid curves illustrate the relationship between predicted probabilities (x-axis) of not achieving the MCID for each WOMAC score one year after total knee arthroplasty and the observed probabilities (y-axis) of these outcomes across the entire dataset. Dashed lines represent perfect agreement between the predicted and observed probabilities **(A)**, WOMAC pain; **(B)**, WOMAC stiffness; **(C)**, WOMAC function; **(D)**, WOMAC total. MCID, minimal clinically important difference; WOMAC, Western Ontario and McMaster Universities Osteoarthritis Index

Table 3 summarizes the performance metrics for the ML models on the test data set. The ExtraTrees model performed best across all performance metrics, particularly excelling in MCE, for all WOMAC subscales and total (Table 3). The AUCs for the best models were 0.92, 0.90, 0.87, and 0.89 for WOMAC pain, stiffness, function, and total, respectively (Fig. 1). At the optimal threshold, recall rates were 0.79, 0.86, 0.70, and 0.83, with specificity around 80% (Table 3). Calibration curves for all WOMAC subscales and total showed good agreement between predicted probabilities and the observed prevalence of not achieving the MCID, with Brier scores of 0.09, 0.10, 0.11, and 0.06 and MCEs of 0.17, 0.14, 0.15, and 0.17, respectively (Fig. 2; Table 3).

**Table 3:** Performance metrics for models predicting not achieving MCIDs at 1 year after total knee arthroplasty on test data set

Variable	Performance metrics	ExtraTrees	Gaussian Naïve Bayes	K-Nearest Neighbors	Logistic Regression	Multi-Layer Perceptron
WOMAC Pain	AUC	0.92	0.85	0.86	0.92	0.91
	Recall	0.79	0.77	0.71	0.76	0.74
	Specificity	0.93	0.76	0.86	0.92	0.94
	Accuracy	0.89	0.77	0.81	0.87	0.88
	Brier score	0.09	0.19	0.13	0.09	0.1
	MCE	0.17	0.41	0.26	0.22	0.23
WOMAC Stiffness	AUC	0.90	0.81	0.80	0.88	0.88
	Recall	0.86	0.74	0.75	0.81	0.77
	Specificity	0.77	0.76	0.72	0.79	0.83
	Accuracy	0.80	0.75	0.73	0.80	0.83
	Brier score	0.10	0.18	0.15	0.11	0.11
	MCE	0.14	0.43	0.26	0.17	0.22
WOMAC Phyiscal Function	AUC	0.87	0.80	0.79	0.84	0.85
	Recall	0.70	0.73	0.76	0.67	0.87
	Specificity	0.85	0.73	0.74	0.84	0.61
	Accuracy	0.82	0.73	0.74	0.80	0.67
	Brier score	0.11	0.19	0.14	0.12	0.14
	MCE	0.15	0.45	0.24	0.24	0.35
WOMAC Total	AUC	0.89	0.85	0.81	0.86	0.86
	Recall	0.83	0.78	0.73	0.71	0.51
	Specificity	0.82	0.78	0.83	0.87	0.94
	Accuracy	0.82	0.78	0.81	0.85	0.88
	Brier score	0.06	0.13	0.08	0.07	0.08
	MCE	0.17	0.64	0.20	0.35	0.41

MCID, Minimal clinically important difference; WOMAC, Western Ontario and McMaster Universities osteoarthritis index; AUC, area under the receiver operating characteristic curve; MCE, maximum calibration error

Given that women comprised 90.3% of the study population, a sex-based subgroup analysis was performed using the best-performing model (ExtraTrees) to explore potential differences in predictive performance (Appendix Table 2). Across all WOMAC subscales and total scores, AUCs were comparable between sexes (0.85–0.95). While recall was higher in men (0.88 vs. 0.78 for WOMAC pain; 0.92 vs. 0.85 for stiffness; 0.87 vs. 0.68 for function; 0.92 vs. 0.81 for total), women showed greater specificity (0.93 vs. 0.87 for WOMAC pain; 0.79 vs. 0.63 for stiffness; 0.87 vs. 0.68 for function; 0.85 vs. 0.53 for total) and better calibration with lower MCEs (0.22 vs. 0.54 for WOMAC pain; 0.22 vs. 0.36 for stiffness; 0.15 vs. 0.34 for function; 0.21 vs. 0.25 for total).

### Model performance: decision curve analysis

**Fig. 3:**
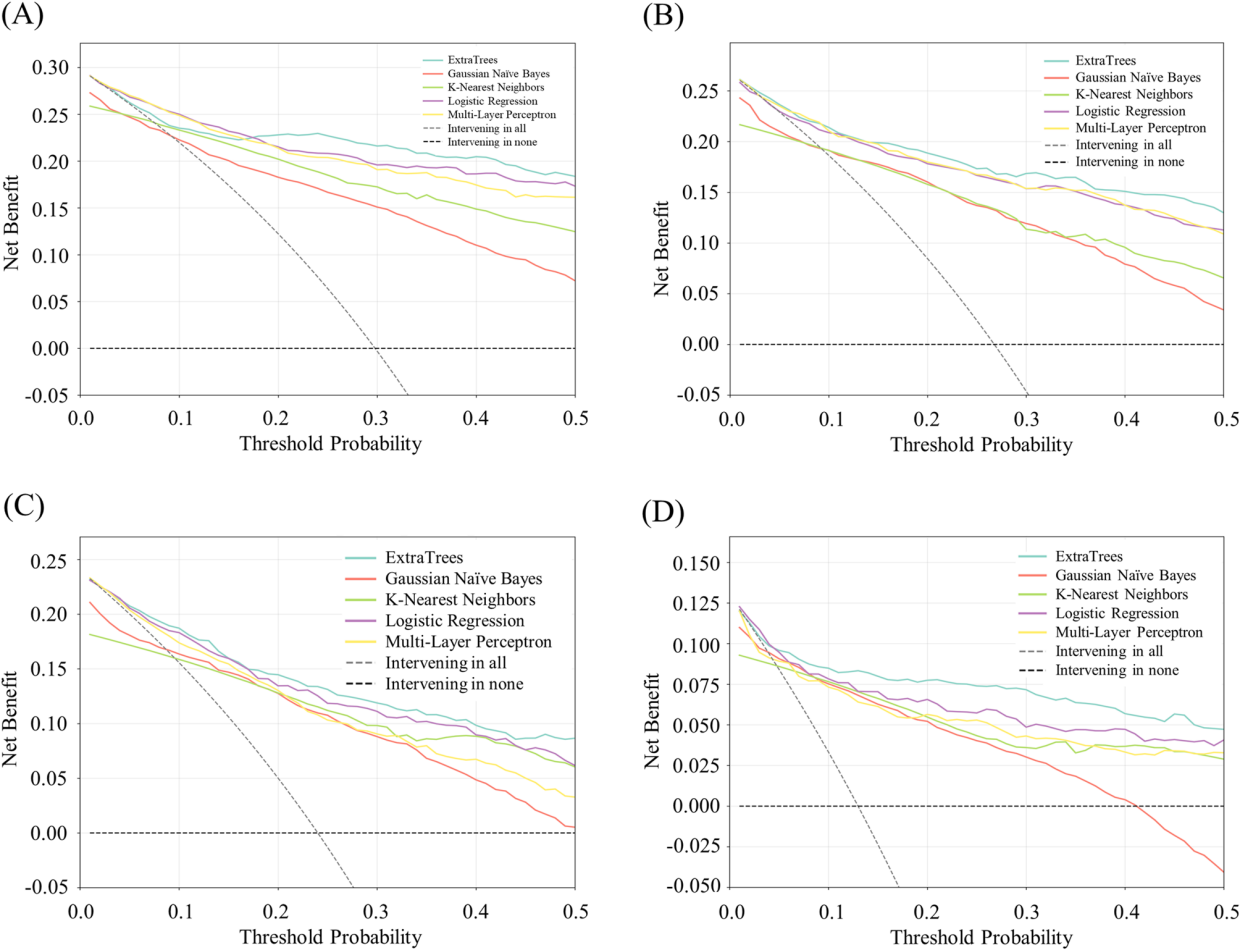
Decision curve analysis for the models. The plots show the net benefit (y-axis) of each machine learning model across a range of threshold probabilities (x-axis) for predicting not achieving MCIDs at 1 year following total knee arthroplasty. Net benefits are compared against two default strategies: intervening in all patients (gray dashed line) and intervening in none (black dashed line). A model is considered clinically useful at the threshold probabilities where its net benefit exceeds both default strategies. **(A)**, WOMAC pain; **(B)**, WOMAC stiffness; **(C)**, WOMAC function; **(D)**, WOMAC total. MCID, minimal clinically important difference; WOMAC, Western Ontario and McMaster Universities Osteoarthritis Index

The clinical utility of the ML models was assessed using DCA (Fig. 3). For all WOMAC subscales and total score, all models demonstrated greater net benefit than the default strategies of “intervening in all” patients or “intervening in none” across a wide and clinically relevant range of threshold probabilities. These findings indicate that using the models to guide intervention decisions would provide greater net benefit than applying a universal management strategy. Among the models, the ExtraTrees model consistently provided the highest net benefit across the majority of thresholds, demonstrating its superior clinical utility.

### Variable importance in the best-performing model

**Fig. 4:**
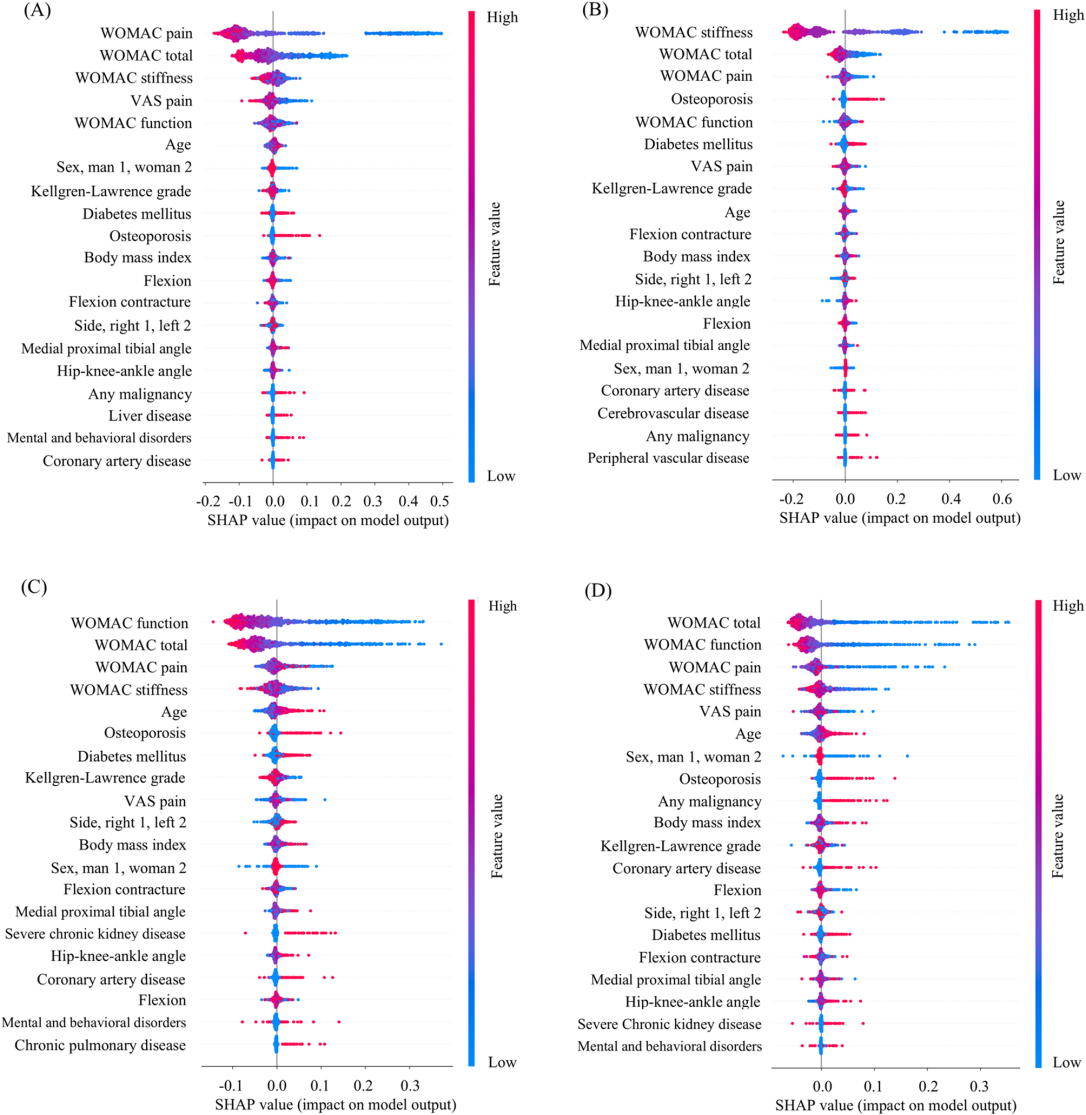
SHAP summary plots for the best-performing models. The plots show each variable’s contribution to the model’s predictions, with variables ranked by importance on the y-axis. Each dot along the horizontal axis represents a SHAP value (x-axis), with colors ranging from blue (low) to red (high). Red dots clustering to the right and blue dots to the left signify an increased risk with rising values of the variable. **(A)**, WOMAC pain; **(B)**, WOMAC stiffness; **(C)**, WOMAC function; **(D)**, WOMAC total. SHAP, Shapley additive explanation; WOMAC, Western Ontario and McMaster Universities Osteoarthritis Index

For all WOMAC subscales and total, the superior preoperative scores were identified as the most important predictor of not achieving the respective MCIDs 1 year after TKA (Fig. 4). Additionally, preoperative values of other WOMAC subscales showed association with insufficient improvements in various subscales (Fig. 4).

Beyond these WOMAC scores, several comorbidities and demographic factors were also associated with insufficient clinical improvement. Osteoporosis was linked to a higher risk of insufficient clinical improvement in WOMAC pain, stiffness, function, and total scores (Fig. 4A-D). Diabetes mellitus was associated with insufficient improvement in WOMAC stiffness and function (Fig. 4B and C). Older age was associated with insufficient improvement in WOMAC function and total (Fig. 4C and D). Malignancy and coronary artery disease showed weak associations with insufficient improvement in WOMAC pain, stiffness, and function, but were more prominently associated with insufficient improvement in WOMAC total (Fig. 4D). Congestive heart failure, arrhythmias, peripheral vascular disease, cerebrovascular disease, chronic pulmonary disease, liver disease, severe chronic kidney disease, and mental and behavioral disorders were not important predictors.

## Discussion

The most important findings of this study were as follows: (1) the ML models demonstrated good discrimination and calibration, as well as net clinical benefit, in preoperatively predicting whether improvements in WOMAC scores 1 year after TKA would not exceed their respective MCIDs; (2) important predictive variables included better preoperative WOMAC scores, osteoporosis, diabetes mellitus, older age, malignancy, and coronary artery disease; and (3) taken together, the models were expected to enhance SDM whether to proceed with TKA and perioperative patient management by preoperatively identifying approximately 70% to over 80% of patients unlikely to achieve MCIDs, with a specificity of about 80%, and providing explanations for the associated factors.

To our knowledge, this is the first study to utilize ML to predict not achieving MCIDs for WOMAC scores following TKA. Among the range of ML algorithms assessed, including traditional methods and advanced non-linear methods, the ExtraTrees model demonstrated the best performance across all WOMAC subscales and total, with superior discrimination, calibration, and clinical utility (Table 3; Figs. 1, 2 and 3). These excellent performances may be attributed to the large, high-quality dataset obtained through standardized perioperative protocols and consistent PROMs monitoring at a single tertiary hospital.

Previous studies employing ML models have predicted whether patients would exceed MCIDs, experience satisfaction, or encounter limitations in walking following TKA [17–24]. While some models achieved promising results for specific subscales, others revealed low AUC values or suboptimal calibration plots, suggesting areas in need of improvement [17–19, 21, 24]. Moreover, aside from a few studies [18], most prior studies predominantly relied on conventional performance metrics for discrimination and calibration (e.g., AUC, Brier score) to evaluate model quality, without accounting for the clinical consequences of a decision. However, high discrimination and calibration alone do not inform clinicians whether using a model to guide patient care would ultimately do more good than harm [40]. In contrast, the present study moves beyond these traditional assessments by evaluating the models’ clinical utility through DCA. As detailed in the Methods section, it quantifies the ‘net benefit’ of using a model by weighing the benefits of true positives (correctly identifying at-risk patients) against the harms of false positives (subjecting low-risk patients to unnecessary interventions by misclassification) across a range of threshold probabilities that reflect clinical decision preferences. Our models—particularly ExtraTrees—demonstrated consistently greater net benefit than the universal management strategies of intervening in all patients or in none (Fig. 3). This finding suggests that our model is not only statistically robust but also clinically valuable when applied to real-world clinical decision-making.

Although earlier research identified predictive variables, explicit connections between these variables and individual subscales [17, 18, 21, 22, 24], as well as their directional effects on outcomes [17, 23], remained undetailed. In the present study, SHAP analysis for each WOMAC subscale and total clarified both the importance of individual variables and whether they had positive or negative relationships with outcomes. For all WOMAC domains, superior preoperative scores in a particular subscale were the most important for predicting insufficient clinical improvement in that same domain after TKA (Fig. 4). This finding is consistent with prior research [12, 14, 15, 19, 21, 41, 42] and can be explained by two reasons: (1) patients who present worse preoperative PROMs generally have greater potential room for improvement; and (2) PROMs are subject to ceiling effects that hinder detection of changes at the higher end of the scoring range [12]. Other preoperative WOMAC subscales also emerged as important for predicting insufficient improvements in different subscale scores, which is likely due to the interrelationships among the subscales despite each addressing a unique dimension. Clinically, for instance, pain and stiffness often present together, or one can influence functional ability, as reported by Clement et al. [43].

Osteoporosis emerged as an important variable for predicting insufficient improvement in WOMAC pain, function, stiffness, and total in the present study. First, in terms of pain, Li et al. [44] identified low bone mineral density as an important predictor of poor improvement in pain after TKA. They suggested that insufficient bone strength to support the prosthesis leads to mechanical instability, and thereby worsens pain [44]. Although aseptic loosening was mentioned as an example [44], it typically occurs at the cement-prosthesis interface rather than the bone-cement interface, making it less relevant in this context [45]. More plausibly, pain may originate from micro-movements between the prosthesis-cement complex and the bone. Second, with regard to function, several studies have reported an association between osteoporosis and worse functional outcomes following TKA [21, 46, 47]. Bone is metabolically active and responds to mechanical stimuli through remodeling and increased density. Given this, patients with impaired physical function are at higher risk for osteoporosis and may also experience limited functional recovery after surgery. This could explain the association observed between osteoporosis and WOMAC function in our study. From another perspective, poor nutrient intake combined with insufficient exercise can result in both muscle weakness and osteoporosis [48]. Muscle weakness contributes to deficits in balance and reduced ability to perform activities [46, 49]. Additionally, the insufficient pain improvement linked to osteoporosis could further impede functional improvement. Third, in relation to stiffness, Ha et al. [50] also found a positive correlation between bone mineral density and WOMAC stiffness. As discussed above, osteoporosis was associated with compromised physical function, which may hinder active participation in postoperative range of motion exercises, potentially increasing stiffness. Moreover, in individuals with osteoporosis, concerns over fracture risk may discourage both active and passive range of motion exercises, further contributing to postoperative stiffness.

As shown in numerous studies [4, 13, 43, 51, 52], diabetes mellitus was an important variable for predicting insufficient improvement in WOMAC stiffness in the current study. The underlying mechanism likely involves the accelerated formation of advanced glycation end products (AGEs) under hyperglycemic conditions [53–55]. AGEs facilitate enhanced collagen cross-linking and promote the production of dense collagen during wound healing, leading to a capsular thickening and increased formation of scar tissue [4, 53], thereby potentially contributing to joint stiffness. Diabetes mellitus was also associated with insufficient functional improvement, which aligns with findings from previous studies [4, 53, 56]. As discussed above, diabetes mellitus is implicated in joint stiffness, which can adversely affect functional ability [57]. Additionally, prior research has identified a relationship between diabetes mellitus and reduced muscle strength in elderly individuals, further contributing to diminished functional capacity [58].

While malignancy and coronary artery disease were not among the higher-ranking predictors of insufficient improvement in WOMAC subscales, they were more prominently associated with insufficient improvement in the WOMAC total. This suggests that these conditions may exert a diffuse and cumulative negative effect across multiple WOMAC subscales, which becomes apparent in the overall WOMAC total. Even when patients have been successfully treated for malignancy and are considered suitable candidates for TKA, long-term sequelae involving musculoskeletal and nervous systems, combined with chronic low-grade systemic inflammation, may negatively impact postoperative pain, stiffness, and function [59–61]. In addition, psychological distress and pain catastrophizing in cancer survivors may contribute to the development of chronic pain following TKA [62]. As for coronary artery disease, the underlying systemic inflammatory environment and microvascular dysfunction including endothelial dysfunction may contribute to increased postoperative pain and impaired tissue healing, resulting in joint stiffness [63, 64]. Furthermore, coronary artery disease was shown to be linked to lower levels of physical activity [65, 66], which may in turn lead to insufficient improvement in WOMAC function, as observed in this study.

As dissatisfaction rates after TKA have decreased from 20 to 10% [9, 67], the predictive challenge has intensified, making it more difficult to achieve the high recall necessary to identify this smaller cohort of at-risk patients without sacrificing the specificity required to avoid misclassifying the successful majority. The models in this study achieved high recall of 70% to over 80% in identifying patients likely to experience insufficient improvement, while attaining specificity at around 80%. This allows for preoperative discussions on expected outcomes and personalized risk, thereby enhancing SDM.

Building upon this predictive insight, patients at high risk of insufficient improvement could benefit from personalized perioperative strategies. For instance, preoperative targeted exercise interventions such as strength, balance, or flexibility training may help enhance postoperative outcomes and recovery [68]. In addition, structured psychosocial interventions, including expectation management and cognitive-behavioral therapy, may improve patient satisfaction and reduce postoperative pain and pain catastrophizing [69–72]. Postoperatively, these patients may be enrolled in more intensive and supervised rehabilitation pathways tailored to their deficits, with the aim of promoting better clinical outcomes [73]. Such applications highlight the potential of our predictive model not only to support SDM regarding whether to undergo TKA, but also to guide the implementation of personalized perioperative interventions aimed at optimizing patient outcomes.

This study had several limitations. First, the MCIDs used to define our primary outcome were adopted from external literature rather than derived from our study population, due to the retrospective nature of our dataset and the absence of anchor-based patient feedback. To address this limitation, we selected thresholds from a high-quality, widely cited study that used anchor-based methods in a TKA cohort with clinical characteristics comparable to ours [32]. Moreover, the proportion of patients not achieving MCID in our cohort (Table 2) was comparable to the widely reported dissatisfaction rate following TKA [8–10], providing indirect support for the clinical relevance of the adopted MCIDs. While adopting externally validated MCIDs is common methodology in orthopaedic research [74], the lack of internal validation remains a methodological limitation. Second, psychological status and socioeconomic factors, which are known to be potential predictors [75, 76], were not available due to the retrospective nature of the study and thus not included in the model. Although we included mental and behavioral disorders (ICD-10 codes F00–F99) as a proxy for psychological status, this variable is less granular than formal patient-reported assessments and likely captures only the more severe spectrum of conditions that require a formal diagnosis. Third, although all patients received standardized postoperative rehabilitation education and monitoring, individual adherence and effort likely varied and were not directly measured. Such unmeasured differences in rehabilitation compliance may have confounded the observed outcomes. Moreover, our dataset did not capture the extent of social support available to each patient after surgery, an important factor that has been shown to influence postoperative recovery and PROMs after TKA [77]. Fourth, the datasets in this study included a high proportion of women (90.3%, Table 1). Given previous reports indicating that women constitute approximately 80–92% of TKA recipients in Asian populations [78–80], along with the initial datasets (*n* = 7,508) in the present study where women comprised 89.3%, the dataset is likely representative and well-sampled. Nevertheless, the limited number of men in the dataset raises concerns regarding the generalizability of the model across sexes. In sex-based subgroup analysis, the model demonstrated comparable AUCs and superior recall in men. However, this higher recall in men was accompanied by worse specificity and calibration, as represented by higher MCE, when compared to the women subgroup. While these findings suggest that the model may still be clinically informative for men, caution is warranted in its interpretation and application. Further model refinement using larger datasets that include a greater number of men may help enhance its robustness and generalizability across sexes. Fifth, given the retrospective nature of this study, prospective research assessing the model’s real-world clinical performance will help strengthen the evidence for its clinical utility. Sixth, external validation of the models was not performed. However, external validation may prove challenging due to institutional differences in perioperative protocols and data collection. Instead, this study demonstrates that an ML model, when utilized with a high-quality dataset, can accurately predict insufficient improvement following TKA.

## Conclusions

The ML models demonstrated good discrimination and calibration, as well as net clinical benefit, in preoperatively predicting insufficient clinical improvement at 1 year after TKA using WOMAC. In these predictions, better preoperative WOMAC scores, osteoporosis, diabetes mellitus, older age, malignancy, and coronary artery disease emerged as important variables. The models have the potential to enhance SDM and perioperative patient management by preoperatively identifying approximately 70% to over 80% of patients likely to experience insufficient clinical improvement, with a specificity of about 80%, and by providing explanations regarding associated factors.

## Supplementary Information

Below is the link to the electronic supplementary material.

Supplementary Material 1

## Data Availability

No datasets were generated or analysed during the current study.

## Abbreviations

TKA: Total knee arthroplasty
SDM: Shared decision-making
ML: Machine learning
WOMAC: Western Ontario and McMaster Universities Osteoarthritis Index
MCID: Minimal clinically important difference
AAOS: American Academy of Orthopaedic Surgeons
PROMs: Patient-reported outcome measures
HKAA: Hip-knee-ankle angle
MPTA: Medial proximal tibial angle
K-L Grade: Kellgren-Lawrence grade
BMI: Body mass index
DCA: Decision curve analysis
MCE: Maximum calibration error
AUC: Area under the receiver operating characteristic curve
SHAP: Shapley additive explanations
AGEs: Advanced glycation end products
