# Supplementary material for: A development of machine learning models to preoperatively predict insufficient clinical improvement after total knee arthroplasty
Source: J Orthop Surg Res. 2025 Aug 20;20:778. doi: 10.1186/s13018-025-06206-z (PMC12366143; doi:10.1186/s13018-025-06206-z)
Supplement: Supplementary file 1 — Supplementary Material 1 [file 13018_2025_6206_MOESM1_ESM.docx]

**Supplementary file (Appendix)**

**Title**

A Development of Machine Learning Models to Preoperatively Predict Insufficient Clinical Improvement After Total Knee Arthroplasty

**Appendix Table 1.** Hyperparameter grid for model tuning across algorithms

| Algorithm | Parameter | Value |
| --- | --- | --- |
| Logistic Regression | C | 0.01, 0.1, 1, 10 |
|  | penalty | l2 |
|  | solver | liblinear, lbfgs |
| K-Nearest Neighbors | N-neighbors | 3, 5, 7 |
|  | weights | uniform, distance |
|  | metric | euclidean, manhattan |
| Gaussian Naïve Bayes | var_smoothing | 1e-9, 1e-8, 1e-7 |
| Multi-Layer Perceptron | hidden_layer_sizes | (50,), (100,), (50, 50) |
|  | activation | relu, tanh |
|  | alpha | 0.0001, 0.001, 0.01 |
|  | learning_rate | constant, adaptive |
| ExtraTrees | n_estimators | 100, 200, 500 |
|  | max_depth | None, 10, 20 |
|  | min_samples_split | 2, 5 |
|  | max_features | sqrt, log2 |

- All models underwent hyperparameter tuning using 5-fold stratified cross-validation.

- Grid search was optimized for the area under the receiver operating characteristic curve (AUC).

- A fixed random state (42) was applied to ensure methodological reproducibility.

- Default values were used for parameters not explicitly listed.

**Appendix Table 2.** Sex-stratified performance of the ExtraTrees model (trained on the combined dataset) in predicting failure to achieve MCIDs 1 year postoperatively on the test data set.

| Variable | Performance metrics | Women | Men |
| --- | --- | --- | --- |
| WOMAC Pain | AUC | 0.92 | 0.95 |
|  | Recall | 0.78 | 0.88 |
|  | Specificity | 0.93 | 0.87 |
|  | Accuracy | 0.89 | 0.88 |
|  | Brier score | 0.09 | 0.07 |
|  | MCE | 0.22 | 0.54 |
| WOMAC Stiffness | AUC | 0.90 | 0.93 |
|  | Recall | 0.85 | 0.92 |
|  | Specificity | 0.79 | 0.63 |
|  | Accuracy | 0.80 | 0.72 |
|  | Brier score | 0.10 | 0.10 |
|  | MCE | 0.22 | 0.36 |
| WOMAC Phyiscal Function | AUC | 0.87 | 0.85 |
|  | Recall | 0.68 | 0.87 |
|  | Specificity | 0.87 | 0.68 |
|  | Accuracy | 0.82 | 0.75 |
|  | Brier score | 0.11 | 0.15 |
|  | MCE | 0.15 | 0.34 |
| WOMAC Total | AUC | 0.89 | 0.90 |
|  | Recall | 0.81 | 0.92 |
|  | Specificity | 0.85 | 0.53 |
|  | Accuracy | 0.85 | 0.59 |
|  | Brier score | 0.06 | 0.08 |
|  | MCE | 0.21 | 0.25 |

MCID, Minimal clinically important difference; WOMAC, Western Ontario and McMaster Universities osteoarthritis index; AUC, area under the receiver operating characteristic curve; MCE, maximum calibration error.
